# Photosymbiotic ascidians in Singapore: turbid waters may reduce living space

**DOI:** 10.3897/zookeys.305.4893

**Published:** 2013-05-30

**Authors:** Shih-Wei Su, Euichi Hirose, Serina Lee Siew Chen, Michael Hin-Kiu Mok

**Affiliations:** 1 Department of Marine Biotechnology and Resources, National Sun Yat-sen University, Kaohsiung 80424, Taiwan; 2 Faculty of Science, University of the Ryukyus, Nishihara, Okinawa 903-0213, Japan; 3 Tropical Marine Science Institute, National University of Singapore, Singapore 119227, Republic of Singapore; 4 Institute of Marine Biology, National Sun Yat-sen University, Kaohsiung 80424, Taiwan

**Keywords:** Algal symbiosis, Ascidian, Biogeography, Coral reefs, Didemnidae

## Abstract

The photosymbiotic ascidian fauna at Changi Beach, Pulau Semakau, Sentosa and St. John’s Island, Singapore were surveyed. A total of five species, *Diplosoma simile*, *Lissoclinum bistratum*, *Lissoclinum punctatum*, *Lissoclinum timorense* and *Trididemnum cyclops*, were recorded, with *Lissoclinum timorense* and *Trididemnum cyclops* being newly recorded in Singapore. However, no photosymbiotic species were found at Changi Beach probably due to the polluted waters in the region. Coastal development has caused Singapore waters to become turbid, leading to decrease in suitable habitats for photosymbiotic ascidians. Clean waters in Pulau Semakau probably provide a better environment for the growth of photosymbiotic ascidians and this area has a greater variety of these ascidians than the other areas in Singapore. Each of the five species has also been recorded in the Ryukyu Archipelago (Japan) and three species (*Diplosoma simile*, *Lissoclinum bistratum* and *Trididemnum cyclops*) have also been recorded in Taiwan.

## Introduction

Photosymbioses have been known in some colonial ascidians of the family Didemnidae in tropical and subtropical waters. Photosymbionts such as *Prochloron* and *Synechocystis* are cyanobacteria (see Parry and Kott 1988; Lewin and Cheng 1989; Hirose et al. 2009b). About 30 species in four didemnid genera were described as host species (Kott 2001). Most of these ascidian hosts always harbor particular cyanobacterial species (i.e., obligate symbiosis), whereas some hosts occasionally harbor the photosymbionts (i.e., facultative symbiosis). Recent taxonomic studies described seven photosymbiotic ascidians from the Ryukyu Archipelago of Japan as new species (Oka et al. 2005; Hirose and Oka 2008; Hirose and Hirose 2009, 2011; Hirose et al. 2009a).

The biogeographic survey of photosymbiotic didemnids in Ryukyus has recorded the current distribution range for each species. To date, at least 20 photosymbiotic species are known to be distributed in Japan, mainly in the Ryukyu Archipelago (Hirose in press, and references therein). The number of species gradually decrease northward in the Ryukyu Archipelago, which ranges from around 24°N to 31°N. Nineteen species have been recorded from the Yaeyama Islands (the southernmost island group: at ca. 24°N), whereas only four species were recorded from Yakushima and Tanegashima (the northernmost islands: at ca. 30°-31°N). Taiwan (21°-25°N) is positioned close to the southernmost island group of the Ryukyu Archipelago (i.e., the Yaeyama Islands), and 10 species in total were recorded from Kenting (southern Taiwan) and Lyudao (off the southeast coast of Taiwan), but no photosymbiotic species were found in Keelung (located in northern Taiwan), probably due to the cold surface water in winter (ca. 16°C) in that region (Hirose and Nozawa 2011; Hirose and Su 2011).

Singapore is positioned at the equator and there are only a few reports on the ascidian fauna in Singapore waters (e.g., Berrill 1950; Millar 1975, 1988). Regarding photosymbiotic ascidians from Singapore, Kott (1982) listed three species, namely *Lissoclinum bistratum*, *Lissoclinum punctatum*, and *Diplosoma simile*. Moreover, occurrence of some other photosymbiotic ascidians was reported as “green gum drops ascidians” awaiting identification (Wild Singapore Homepage, http://www.wildsingapore.com/wildfacts/ascidiacea/greengumdrop.htm). In view of the numbers of species recorded in Japan and Taiwan, more photosymbiotic species were expected to be found in Singapore.

During 2009-2011, we surveyed the photosymbiotic ascidians at Changi Beach, Pulau Semakau, Sentosa and St. John’s Island, Singapore in collaboration with the Tropical Marine Science Institute, National University of Singapore. Herein, five photosymbiotic didemnid species were reported as additions to the marine benthic fauna of Singapore.

## Material and methods

**Figure 1.**
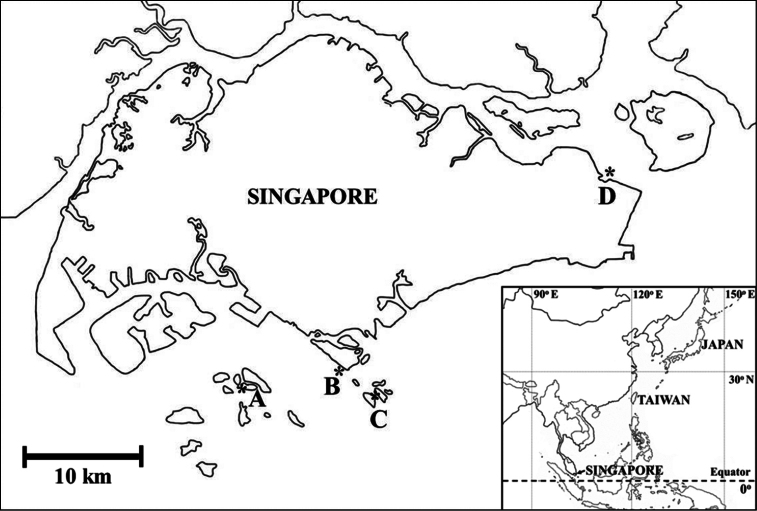
Collection sites of photosymbiotic ascidians in Singapore. **A** Pulau Semakau **B** Sentosa **C** St. John‘s Island and **D** Changi Beach.

Ascidian colonies were collected by snorkeling in the shallow subtidal zone. Samples were photographed *in situ* before being collected. Collection sites were as follows: Changi Beach, Pulau Semakau, Sentosa, and St. John’s Island (Fig. 1). The habitats consisted of sand beach, seagrass meadow, coral rubble, sheltered beach and coral reefs. The Changi Beach is a beach park located at the northeastern of Singapore. The park is approximately 3.3 km long with stretches of sandy beaches and a lot of seagrass. Pulau Semakau is located at the south of the main island of Singapore. There is a vast seagrass meadow and a wide zone of coral rubble with various marine lives and an enormous area rich in wildlife. Sentosa is a popular island resort in Singapore that includes a 2 km long sheltered beach. St. John’s Island is one of the Southern Islands in Singapore. The hilly island is transformed into a tranquil getaway. Coral reefs and seagrass bed scatter in the zone. Specimens were anesthetized with menthol and 0.37 M MgCl_2_ for approximately 2 h, and then fixed with 10% formalin-seawater. The fixed colonies were dissected under a binocular stereomicroscope and a compound microscope equipped with differential interference contrast optics. In some photomicrographs, several images were combined to increase the depth of field using the post-processing image software Helicon Focus Pro 4.2.2 (Helicon Soft, Ltd., Kharkov, Ukraine). Taxa were mainly identified following Kott (2001), Monniot and Monniot (2001), and Hirose and Su (2011). All of the specimens examined were deposited in the Raffles Museum of Biodiversity Research, Singapore (RMBR) or some of those were deposited in the National Museum of Natural Science, Taiwan (NMNS).

## Results

The present report describes the occurrence of didemnid ascidians harboring prokaryotic algae from Pulau Semakau, Sentosa and St. John’s Island, Singapore. In total, five species were collected, two of which were new records for Singapore. Their occurrences at each site and dates are listed in Table 1. No photosymbiotic species were found at Changi Beach.

**Table 1.** Distribution records of photosymbiotic didemnid ascidians in Singapore.<br/>

**Location**	**Changi Beach**	**Pulau Semakau**			**St. John’s Island**		**Sentosa**
**Date**	**2011 Nov**	**2009 Dec**	**2010 May**	**2011 Nov**	**2010 May**	**2011 Nov**	**2010 April**
*Trididemnum cyclops*					+		
*Diplosoma simile*			+	+	+		+
*Lissoclinum bistratum*		+	+	+			+
*Lissoclinum punctatum*			+	+	+		
*Lissoclinum timorense*		+	+	+			

### Trididemnum cyclops

Michaelsen, 1921

Trididemnum symbioticum  (Peres, 1962)

#### Specimens examined.

ZRC-TUN-0012 (St. John’s Island, subtidal at depth 0.5 m).

**Figure 2.**
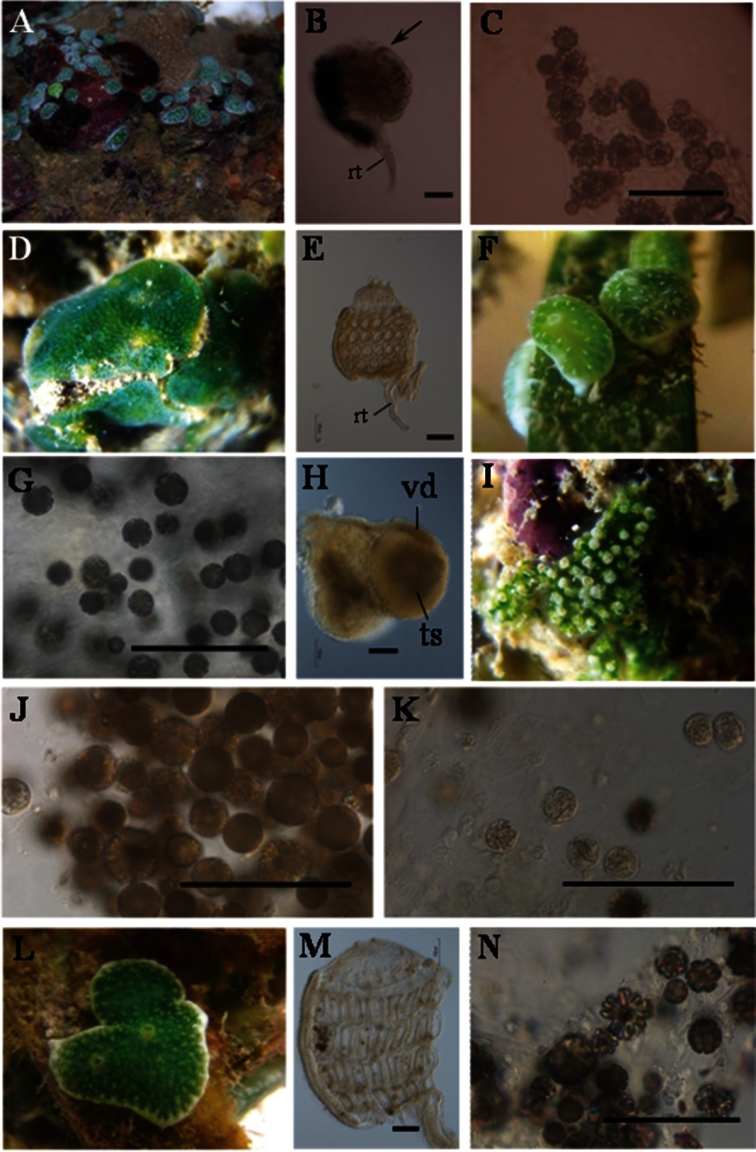
Photosymbiotic ascidians collected. **A** *Trididemnum cyclops*, St. John’s Island, Singapore (Depth = 0.5 m). Colonies are 2–6 mm on the long axis **B** Thorax of *Trididemnum cyclops*. Arrows indicatethe endostylarpigment cap. Scale bar = 100 mm. **C** Tunic spicules in the tunic of *Trididemnum cyclops*. Scale bar = 100 mm **D** *Diplosoma simile*, Pulau Semakau, Singapore (Depth = 0.5 m). Colonies are approximately 15 mm in diameter **E** Thorax of *Diplosoma simile* (left view). Scale bar = 100 mm **F** *Lissoclinum bistratum*, Pulau Semakau, Singapore (Depth = 0.5 m). Colonies are approximately 5 mm in diameter **G** Tunic spicules in the tunic of *Lissoclinum bistratum*. Scale bar = 100 mm **H** A testis with an uncoiled vas deferens of *Lissoclinum bistratum*. Scale bar = 100 mm **I** *Lissoclinum punctatum*, Pulau Semakau, Singapore (Depth = 0.5 m). Colonies are approximately 10 mm in diameter **J** Tunic spicules in the tunic of *Lissoclinum punctatum*. Scale bar = 100 mm **K** Tunic phycocytes of *Lissoclinum punctatum*. Scale bar = 100 mm **L** *Lissoclinum timorense*, Pulau Semakau, Singapore (Depth = 0.5 m). Colonies are approximately 10 mm in diameter **M** Thorax of *Lissoclinum timorense* (left view) **N** Tunic spicules in the tunic of *Lissoclinum timorense*. Scale bar = 100 mm. rt, retractor muscle; ts, testis; vd, vas deferens.

Colonies are oval or irregularly shaped cushions of 2-6 mm on the long axis (Fig. 2A). Each zooid has a black dot, due to a pigment mass at the top of the endostyle (Fig. 2B). Berry-like spicules are distributed in the colonial margin and basal tunic, while they are rarely found in the surface tunic. Spicules are up to 40 mm in diameter (Fig. 2C). The biased distribution of the spicules allows the symbionts to receive sunlight for photosynthesis.

### Diplosoma simile

(Sluiter, 1909)

Diplosoma midori (Tokioka, 1954)
Leptoclinum midori Tokioka, 1954
Leptoclinum simile Sluiter, 1909

#### Specimens examined.

NMNS-7027-001, NMNS-7027-002, ZRC-TUN-0001 and ZRC-TUN-0015 (Pulau Semakau, subtidal at depth 0.5 m), ZRC-TUN-0011 (St. John’s Island, subtidal at depth 0.5 m), ZRC-TUN-0009 (Sentosa, subtidal at depth 0.5 m)

Colonies are irregularly shaped sheets about 2 mm thick without spicules (Fig. 2D). They are entirely green due to the *Prochloron* cells in the common cloacal cavities. The thorax has four stigmatal rows: there are six stigmata in the first (top), second, and third row and five stigmata in the fourth row (bottom). The retractor muscle emerges from the bottom of the thorax (Fig. 2E). Testis and/or egg are found in some zooids, and vas deferens is uncoiled. Kott (1982) reported this species from Singapore. This is one of the most common species in the didemnid-*Prochloron* obligate symbioses in the tropical Pacific, and it has also been recently found in Caribbean Panama (Hirose et al. 2012).

### Lissoclinum bistratum

(Sluiter, 1905)

Didemnum bistratum Sluiter, 1905
Didemnum gottschaldti Tokioka, 1950
Didemnum pulvinum Tokioka, 1954
Leptoclinum bistratum (Sluiter, 1905)
Lissoclinum pulvinum (Tokioka, 1954)

#### Specimens examined.

NMNS-7027-003, NMNS-7027-004, ZRC-TUN-0002, ZRC.TUN.0004, ZRC-TUN-0007, ZRC-TUN-0008 and ZRC-TUN-0014 (Pulau Semakau, subtidal at depth 0.5 m), ZRC-TUN-0010 (Sentosa, subtidal at depth 0.5 m).

Colonies are oval cushions of 4 mm on the long axis (Fig. 2F). The photosymbiont *Prochloron* gives the colonies a green color, while the colonial margin and bottom are white due to dense aggregations of globular spicules in the tunic (Fig. 2G). The thorax has four stigmatal rows. It is difficult to count accurately the number of stigmata owing to the distortion of thoraxes caused by the shrinkage of zooids upon fixation. There are about seven stigmata in each row. Some zooids have a testis with an uncoiled vas deferens (Fig. 2H). Kott (1982) reported this species from Singapore.

### Lissoclinum punctatum

Kott, 1977

#### Specimens examined.

NMNS-7027-007, ZRC-TUN-0005 and ZRC-TUN-0016 (Pulau Semakau, subtidal at depth 0.5 m), ZRC-TUN-0013 (St. John’s Island, subtidal at depth 0.5 m)

Colonies are irregularly shaped sheets about 2 mm thick (Fig. 2I). Globular spicules aggregate around each zooid, which is enclosed in a capsule of white spicules (Fig. 2J). Many *Prochloron* cells are distributed in both cloacal cavities and tunic cells (tunic phycocytes) (Fig. 2K; also see Hirose et al. 1996). We could not examine the zooids in further detail because of the shrinkage of the specimens. Kott (1982) reported this species from Singapore.

### Lissoclinum timorense

(Sluiter, 1909)

Didemnum timorensis Sluiter, 1909
Didemnum voeltzkowi Michaelsen, 1920
Lissoclinum timorensis (Sluiter, 1909)
Lissoclinum voeltzkowi (Michaelsen, 1920)

#### Specimens examined.

NMNS-7027-005, NMNS-7027-006, ZRC-TUN-0003, ZRC-TUN-0006 and ZRC-TUN-0017 (Pulau Semakau, subtidal at depth 0.5 m)

Colonies are irregularly shaped sheets about 2–5 mm thick (Fig. 2L). The colonies are green due to *Prochloron* cells distributed in the common cloacal cavities, while the colonial margin and bottom are white due to the dense distribution of stellate and globular spicules. In the five zooids we examined, the thorax had four stigmatal rows: there were seven stigmata in the first row (top), eight in the second row, seven in the third row, and five or six in the fourth row (bottom) (Fig. 2M). Gonads are not found in the present specimens. There are globular spicules in the tunic (Fig. 2N). The presence of stellate spicules easily distinguishes the present species from *Lissoclinum bistratum*, which lacks these spicules. However, Monniot and Monniot (2001) proposed to regard *Lissoclinum timorense* as a junior synonym of *Lissoclinum bistratum*, because the two species differ only in the shape of spicules and there are no distinctive features in the zooids and larvae. The phylogenetic trees established using the partial sequences of cytochrome oxidase subunit I gene did not discriminate the two species, which are distinguished only by the spicule shapes (Hirose et al. 2010).

## Discussion

Five photosymbiotic ascidians were recorded in the present survey, including three species previously observed by Kott (1982) and two new records in Singapore. There were four species in Pulau Semakau, three species on St. John’s Island and two species in Sentosa, but no photosymbiotic species were found at Changi Beach. The five species listed here might be far from the entire coverage of the photosymbiotic ascidian fauna in Singapore, because the present survey was conducted over a very short period of time and at only four sites. It is expected that more species still remain to be recorded.

Once, there were over 60 offshore islands and patch reefs around Singapore, most of which were situated south of mainland Singapore. However, since the mid 1970s, Singapore has been undergoing coastal reclamation. As its population grows until more than four million, the Singapore government faces problem in providing ample land. Some of offshore islands in Singapore have been deformed or enlarged by some coastal reclamation projects. Many of the coral reef organisms were smothered by reclamation, while others were severely affected by the resulting increase in water turbidity. The high turbidity of waters restricts light penetration, and determines the maximum distribution depth for corals and photosymbiotic ascidians. Visibility has been reduced from 10 m in the 1960s to 2 m or less to date (*Coral Reefs of Singapore*, http://coralreef.nus.edu.sg/). As a result, up to 60% of the live coral cover has been lost in Singapore since 1986 (Chou 2006). We had recorded three species (*Trididemnum cyclops*, *Diplosoma simile* and *Lissoclinum punctatum*) at St. John’s Island in May 2010, but since then we were unable to find any photosymbiotic ascidians at the island. There were also two species of photosymbiotic ascidians, *Diplosoma simile* and *Lissoclinum bistratum*, in Sentosa. Interestingly, no photosymbiotic species were found at Changi Beach in the present survey, although its latitude is comparable with that of Pulau Semakau. Although these results do not conclusively demonstrate the absence of photosymbiotic ascidians at Changi Beach, photosymbiotic species must be rare there.

The coastal environment of Singapore is limited and currently severely affected by coastal development and the port industry, which is one of the biggest economic businesses in the country. Harbor limits occupy most of the territorial waters, and reclamation has transformed considerably almost the entire southern and northeastern coasts of the main island (Chou and Goh 1998). Most of the coastal waters are filled with suspended particles that block photosynthetic activities of marine organisms. When these particles sink, they settle over sessile organisms, such as photosymbiotic ascidians, and adversely affect their metabolism and growth (Dionisio-Sese et al. 2001). In 1999, when the last remaining landfill on Singapore’s mainland was exhausted, the Semakau Landfill was created by enclosing Pulau Semakau and a small adjacent island (Pulau Sakeng) with a rock bund. However, the original Pulau Semakau, which was not affected by the landfill construction, has an enormous intertidal area rich in amazing wildlife. There is a wide zone of coral rubble with various marine lives, leading to the coral reefs that line the edge of the island. Clean waters in Pulau Semakau provide a better environment for the growth of photosymbiotic ascidians that are able to perform both photosynthesis and suspension feeding. In Pulau Semakau, we recorded four species of photosymbiotic ascidians, namely, *Diplosoma simile*, *Lissoclinum bistratum*, *Lissoclinum punctatum* and *Lissoclinum timorense* and it is the area in Singapore with the greatest variety of photosymbiotic ascidians.

More than 20 photosymbiotic species are known to be distributed in Japan, mainly in the Ryukyu Archipelago (Hirose in press, and references therein) and 10 species in total were recorded in Taiwan (Hirose and Nozawa 2011, Hirose and Su 2011) (Table 2). Only five species were recorded in Singapore, and each of them had also been recorded in Japan. Among them, *Diposoma simile*, *Lissoclinum bistratum* and *Trididemnum cyclops* were also recorded in Taiwan. The climate of Singapore is typically wet equatorial, with high temperature and high annual precipitation. The average sea surface temperature is about 29°C. The optimum temperature for *Prochloro* n is 35° to 40°C, as the temperature range for proper photosynthesis is between 20°C and 45°C (Dionisio-Sese et al. 2001). Alberte et al. (1986) also showed that photosynthesis of *Prochloron* was fairly sensitive to temperature; the photosynthetic activity at 25°C is only half of that at 30°C, and it falls almost to 0 at 20°C. This sensitivity of *Prochloron* to low temperature may be a prime factor limiting the distribution of this species to tropical waters.

**Table 2.** Distribution records of photosymbiotic didemnid ascidians in Japan, Taiwan and Singapore.<br/>

**Location**	**Japan**	**Taiwan**	**Singapore**
*Didemnum molle*	+	+	
*Diplosoma aggregatum*	+	+	
*Diplosoma gumavirens*	+	+	
*Diplosoma ooru*	+	+	
*Diplosoma simile*	+	+	+
*Diplosoma simileguwa*	+	+	
*Diplosoma variostigmatum*	+		
*Diplosoma virens*	+	+	
*Diplosoma watanabei*	+		
*Lissoclinum bistratum*	+	+	+
*Lissoclinum midui*	+		
*Lissoclinum patella*	+		
*Lissoclinum punctatum*	+		+
*Lissoclinum timorense*	+		+
*Lissoclinum triangulum*	+		
*Trididemnum clinides*	+	+	
*Trididemnum cyclops*	+	+	+
*Trididemnum miniatum*	+		
*Trididemnum nubilum*	+		

(Hirose and Nozawa 2011; Hirose and Su 2011; Hirose in press, and references therein)

## Supplementary Material

XML Treatment for Trididemnum cyclops

XML Treatment for Diplosoma simile

XML Treatment for Lissoclinum bistratum

XML Treatment for Lissoclinum punctatum

XML Treatment for Lissoclinum timorense
